# The efficacy and safety of spesolimab in patients with generalized pustular psoriasis flares: a systematic review and meta-analysis

**DOI:** 10.3389/fmed.2025.1749320

**Published:** 2026-01-15

**Authors:** Dhaii Alzahrani, Afnan Hasanain, Renad Alharthy, Yara Aljefri, Yara Alghamdi, Renad Alshaikh, Almaha Alhijab, Faris Neazy, Abdulmajeed Alosaimi, Badr Felemban, Reshale Johar, Roaa E. Morya

**Affiliations:** 1 College of Medicine, King Saud bin Abdulaziz University for Health Sciences, Jeddah, Saudi Arabia; 2 King Abdullah International Medical Research Center, Jeddah, Saudi Arabia; 3 Department of Dermatology, King Faisal Specialist Hospital and Research Centre, Jeddah, Saudi Arabia; 4 Department of Dermatology, Jeddah Health Cluster, Jeddah, Saudi Arabia; 5 Department of Dermatology, Ministry of the National Guard—Health Affairs, Jeddah, Saudi Arabia; 6 College of Medicine, Arabian Gulf University, Manama, Bahrain

**Keywords:** generalized pustular psoriasis (GPP), IL-36 receptor antagonist, meta-analysis, randomized controlled trials, spesolimab, systematic review

## Abstract

**Background:**

Generalized pustular psoriasis (GPP) is a rare, auto-inflammatory skin disease characterized by unpredictable flares, often requiring hospitalization, and current therapies lack robust evidence.

**Objectives:**

To systematically evaluate the efficacy and safety of spesolimab for acute GPP flares.

**Methods:**

We searched Medline, Google Scholar and the Cochrane Central Register of Controlled Trials from inception to December 2024 and consulted ClinicalTrials.gov and the ISRCTN registry for ongoing studies. Randomized controlled trials enrolling adults experiencing GPP flares and comparing spesolimab with placebo or standard care were eligible. Non-randomized studies, studies without placebo comparators, or without full-text availability were excluded. Two reviewers independently assessed risk of bias using the Cochrane RoB 2 tool and extracted data. A random-effects model was used to pool odds ratios or mean differences with 95% confidence intervals.

**Results:**

Four randomized controlled trials with 176 participants met the inclusion criteria. Compared with placebo, spesolimab significantly increased the proportion of patients achieving complete pustular clearance [GPPGA pustulation score 0; OR 4.87 (95% CI 2.03–11.68)] and a total GPPGA score of 0 or 1 [OR 5.68 (95% CI 2.27–14.23)]. Adverse event rates were similar between groups and were mostly mild or moderate.

**Limitations:**

Evidence was limited by the small number and sample size of trials, heterogeneity between studies and short follow-up durations.

**Conclusion:**

Spesolimab provides clinically meaningful improvements in pustular clearance and symptom control during acute GPP flares and has a favorable safety profile; however, further large, long-term randomized trials are needed.

**Systematic review registration:**

https://www.crd.york.ac.uk/PROSPERO/view/CRD42025629994, identifier CRD42025629994.

## Introduction

Generalized pustular psoriasis (GPP) is a rare, potentially fatal auto-inflammatory dermatosis marked by recurrent episodes of extensive erythema and the formation of sterile pustules (1). The disease affects between 1.76 and 91 people per million people around the world, and it is common in Asian populations (2, 3). The clinical course of GPP is unpredictable, with acute exacerbations instigated by infections, stress, medication withdrawal, or pregnancy (3, 4).

Flares present with systemic inflammation, characterized by fever, malaise, and leukocytosis, often accompanied by debilitating extracutaneous manifestations such as arthritis or hepatobiliary abnormalities (5). Over 50% of patients experiencing flares necessitate inpatient care, indicative of the disease’s severity and systemic characteristics (6). Moreover, the physical and psychological distress experienced during disease exacerbations significantly reduces health related quality of life (7).

Therapeutic management of GPP remains challenging. Traditional systemic agents such as cyclosporine, acitretin, and methotrexate are used off-label but have limited evidence of efficacy from previous controlled trials (8). Biologics that target tumor necrosis factor (TNF), interleukin (IL)-17, and IL-23 pathways are helpful for plaque psoriasis, data supporting their use in GPP are limited to small, open-label, and have heterogeneous designs (9). Until recently, there was no standardized evidence-based treatment specifically authorized for GPP outside of certain East Asian markets, indicating a significant unmet clinical requirement (10).

The pathophysiology of GPP is primarily driven by uncontrolled interleukin-36 (IL-36) signaling, which triggers keratinocyte activation, cytokine release, and neutrophilic infiltration of the epidermis (11). Mutations in the IL36RN gene are observed in up to 75% of patients and result in constitutive IL-36 activation (12). Available genetic and immunologic data support a central role for IL-36 signaling in GPP pathogenesis and provide a biological rationale for therapies that inhibit IL-36 receptor–mediated inflammation.

Spesolimab is a humanized monoclonal antibody that stops the IL-36 receptor from functioning, which stops inflammatory signaling from happening (12). Early clinical studies reported rapid improvements in pustulation and systemic features after IL-36 receptor blockade; however, estimates of effect vary across study designs and populations, and the comparative evidence base is concentrated in a limited number of randomized datasets (13). The crucial Effisayil 1 randomized controlled trial further illustrated that intravenous spesolimab attained complete pustular clearance (GPP Physician Global Assessment pustulation score of 0) in 54% of participants compared to 6% on placebo after 1 week (4, 9). Following results from randomized trials, spesolimab received regulatory approval for the treatment of GPP flares in multiple regions (3, 10). Nonetheless, existing evidence continues to be disjointed. The majority of data is derived from singular pivotal trials and regional subgroup analyses, constraining the applicability of findings across diverse ethnicities, dosing regimens, and disease severities (13). Moreover, disparities in placebo response, safety event rates, and study design hinder direct interpretation of comparative efficacy (3).

This systematic review and meta-analysis synthesizes randomized evidence on the efficacy and safety of spesolimab for acute GPP flares and summarizes remaining uncertainties relevant to clinical decision-making and future research. The conclusions drawn from this study will assist clinicians and policymakers in refining treatment strategies for this uncommon yet serious disease.

## Materials and methods

### Protocol and registration

This systematic review was conducted in accordance with a pre-specified protocol registered in International Prospective Register of Systematic Reviews (PROSPERO) (CRD42025629994) and reported following the Preferred Reporting Items for Systematic Reviews and Meta-Analyses (PRISMA) guidelines (14).

### Search strategy

A comprehensive literature search was conducted in PubMed, Web of Science, Scopus, and the Cochrane Central Register of Controlled Trials (CENTRAL), without restrictions on language or publication date. The final search was completed in December 2025. Ongoing or recently completed trials were identified through ClinicalTrials.gov and the ISRCTN registry. The following Medical Subject Headings (MeSH) and keywords were used in all data bases: (“Interleukin-36 Receptor Antagonists” AND Spesolimab) AND (“Psoriasis” OR “Pustular”), applying a randomized controlled trial filter. All retrieved records were managed and screened using Rayyan.AI software.

### Study selection criteria

Studies were eligible if they met the following inclusion criteria: (1) participants aged 18–75 years with a moderate-to-severe flare of GPP, defined by a Generalized Pustular Psoriasis Physician’s Global Assessment (GPPGA) total score ≥ 3, new or worsening pustules, a GPPGA pustulation sub-score ≥ 2, and ≥ 5% body surface area affected by erythema and pustules; (2) randomized controlled trial (RCT) design studies;(3) intervention using any available dose of spesolimab; (4) inclusion of a placebo comparator group; and (5) full-text availability. Articles not meeting these criteria were excluded. The PICO framework was used to guide the eligibility process (Table 1).

**TABLE 1:** Population, intervention, comparator, outcomes, and study designs (PICOS) structure.

Study	Population	Intervention	Comparison	Stated outcome/findings
Morita et al. (2)	29 Asian patients with acute GPP flare	Spesolimab 900 mg IV single dose (*n* = 16). Rescue Day-8 dose.	Placebo (*n* = 13)	Higher pustule clearance at Week 1 (62.5% vs. 7.7%; RD 54.8, 95% CI 17.3–79.8) and improved GPPGA total score (50% vs. 15.4%). Rapid improvements in patient reported outcomes such as pain, fatigue, DLQI*, and PSS*with MCID* reached by Week 1–2. Inflammatory markers normalized within 1–2 weeks. Responses sustained through Week 12.
Tsai et al. (1)	11 Chinese patients with acute GPP flare	Spesolimab 900 mg IV single dose (*n* = 5). Rescue Day-8 dose	Placebo (*n* = 6)	Higher rates of pustule clearance at Week 1 (60% vs. 16.7%; RD 43.3%, 95% CI –22.6 to 86.2) and improved GPPGA total score (60% vs. 16.7%). Rapid separation of response curves within the first week, with sustained effects through Week 12. Improvements seen in GPPASI*, pain VAS*, fatigue, DLQI*, and PSS* up to Week 12. Safety similar between groups, mostly mild–moderate Adverse events (Aes); one skin SCC* occurred after two doses of open-label spesolimab and was assessed as unrelated.
Bachelez et al. (4)	53 global adults with acute GPP flare	Spesolimab 900 mg IV single dose (*n* = 35). Rescue Day-8 dose.	Placebo (*n* = 18)	Rapid pustule clearance at Week 1 (54% vs. 6%; RD 49%, 95% CI 21–67) and improved GPPGA total score (43% vs. 11%; RD 32%, 95% CI 2–53). Improvements in GPPASI*, pain VAS*, DLQI*, PSS*, FACIT-Fatigue, neutrophil counts, and CRP* were sustained through week 12. Safety was mostly mild–moderate AEs*; infections occurred in 47% of patients, serious AEs in 12%, and two cases of possible DRESS*; antidrug antibodies were detected in 46%.
Morita et al. (3)	123 adults with GPP in remission or low disease activity	Spesolimab 300 mg or 600 mg SC every 12 weeks for 48 weeks: low dose (*n* = 31) medium dose (*n* = 31) high dose (*n* = 30)	Placebo (*n* = 31)	High-dose spesolimab reduced the risk of GPP flares over 48 weeks (10% vs. 52% in placebo; RD -0.39, 95% CI -0.62 to -0.16; *p* = 0.0013) with sustained benefit in patients with and without IL36RN mutations. Improvements were seen in PSS and DLQI scores across all doses. Safety was mostly mild–moderate AEs, similar across doses; serious AEs occurred in 10% of spesolimab patients versus 3% of placebo, with no deaths.

*DLQ1, Dermatology Life Quality Index; *PSS, Psoriasis Symptoms Scale; *MCID, Minimal Clinically Important Difference; *GPPASI, Generalized Pustular Psoriasis Area and Severity Index; *Pain VAS, Pain Visual Analog Scale; *Skin SCC, Skin Squamous Cell Carcinoma; *FACIT-Fatigue, Functional Assessment of Chronic Illness Therapy; *CRP, C-Reactive Protein; AEs, Adverse Effects; DRESS, Drug Reaction with Eosinophilia and Systemic Symptoms.

### Study screening

**FIGURE 1:**
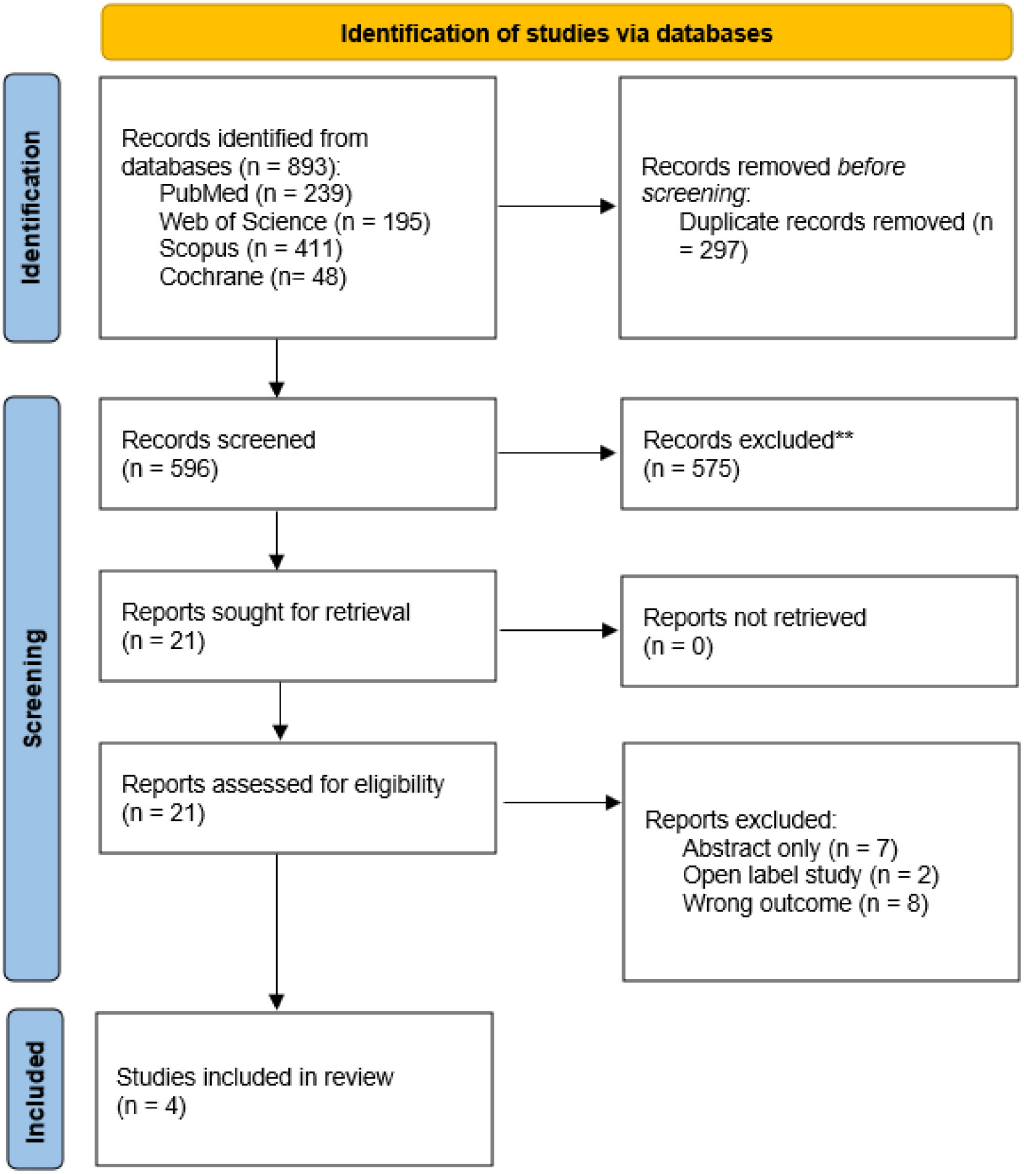
PRISMA flow diagram of included studies.

Two reviewers independently screened titles, abstracts, and full-text articles after removing duplicates. Disagreements were resolved through discussion or consultation with a third reviewer. Most exclusions resulted from non-randomized study designs or insufficient outcome data. A total of 893 records were identified, of which 297 were duplicates and 575 were excluded after title and abstract screening. 21 full-text articles were assessed for eligibility, and four RCT datasets were included in the quantitative synthesis (Effisayil 1 trial) (4), two subgroup analyses (1, 2) derived from the Effisayil 1 trial; as a result, some participants may overlap across datasets; therefore, pooled estimates should be interpreted with caution as they may overweight the Effisayil 1 population and overstate precision, and the Effisayil 2 trial (3). All trials evaluated spesolimab for the treatment or prevention of flares in GPP (Figure 1). Non-randomized evidence (compassionate-use cohorts, retrospective real-world studies, and case series) evaluating spesolimab for acute GPP flares is summarized in Supplementary Table 1.

### Data extraction and management

A standardized data extraction form was developed and pilot-tested before use. Two reviewers independently extracted relevant data from each included study, including study characteristics, participant demographics, intervention details, outcomes, and safety data. Any discrepancies were resolved by a third reviewer.

### Risk of bias assessment

The risk of bias for each included RCT was independently evaluated by two reviewers using the Cochrane Risk of Bias 2 (RoB 2) tool (15). In addition, the Robvis visualization tool was used to create risk of bias figures (16). The five domains assessed included: (Domain 1) bias arising from the randomization process, (Domain 2) deviations from intended interventions, (Domain 3) missing outcome data, (Domain 4) measurement of the outcome, and (Domain 5) selection of the reported result. Any disagreements were resolved through discussion or adjudication by a third reviewer.

### Statistical analysis

Statistical analyses were conducted using Review Manager (RevMan) version 5.4 (Cochrane Collaboration). Effect sizes were calculated using odds ratios (ORs) with 95% confidence intervals (CIs). Meta-analyses were performed using a random-effects model, given expected clinical heterogeneity among studies. The co-primary efficacy outcomes were (1) achievement of GPPGA pustulation sub-score 0 at Week 1 and (2) achievement of GPPGA total score 0 or 1 at Week 1. Secondary outcomes included any adverse event and other outcomes reported by trials. Outcomes were prespecified in the protocol. Statistical heterogeneity was assessed using the I^2^ statistic, with values > 50% indicating substantial heterogeneity. No further data conversions or imputations were carried out; all analyses utilized the summary statistics provided directly in the included studies. To address potential heterogeneity among study results, randomized controlled trials exhibiting high clinical or methodological heterogeneity were excluded from the meta-analysis. Subgroup analyses and meta-regression were not conducted due to the limited number of included studies. Sensitivity analyses were conducted to assess the robustness of the synthesized results by excluding the RCTs that contributed most to heterogeneity. The effect estimates from these analyses were compared with the primary meta-analysis to confirm the stability and reliability of the findings. Forest plots were used to visually present the results of meta-analyses.

### Reporting bias

Due to the limited number of included studies, formal assessment of publication bias using funnel plots or statistical tests were not conducted.

### Certainty of evidence

GRADE assessment was done by two independent researchers. The GRADEpro GDT tool was used to evaluate the certainty of evidence for each outcome (17). Certainty was judged for each outcome across five domains: risk of bias, inconsistency, indirectness, imprecision and publication bias.

## Results

### Study characteristics

This systematic review included four RCTs conducted across 32 countries in North America, Europe, and Asia (1–4). Three trials followed participants for 12 weeks, while one had a 48 weeks follow-up. Sample sizes ranged from 18 to 92, and the studies were published between 2021 and 2023. All trials investigated spesolimab, but differed slightly in administration method, dosage, frequency, and duration. Three of the studies administered spesolimab 900 mg intravenously, while one study used subcutaneous injections of spesolimab in three different doses of 600, 300, and 150 mg. In total, 176 participants aged 18–75 years were included, with a predominance of females (62–72%) and Asian participants (64%). At baseline, disease severity was moderate to severe, reflected by mean GPPGA scores of 3 or higher (Table 2).

**TABLE 2:** Characteristics of included studies.

Study	Country	Follow-up time	Experimental	Control	Number of patients		Gender		Age in years (mean ± SD)		Research design	Inclusion of population
					Experimental	Control	Male	Female	Experimental	Control		
Morita et al. (3)	Multinational (20 countries)	48 weeks	Low dose spesolimab: SC 300 mg → 150 mg q12w; Medium dose spesolimab: SC 600 mg → 300 mg q12w; High dose spesolimab: SC 600 mg → 300 mgq4w	Placebo	92	31	11 (35%)	20 (65%)	Low dose: 38.9 (16.5) Medium dose: 42.9 (16.7) High dose: 40.2 (16.4)	39.5 (14.0)	Randomized, double-blind, placebo–controlled (Effisayil 2)	Patients aged 12–75 years with a documented history of GPP (per ERASP EN* criteria), ≥ 2 past flares, and GPPGA 0–1 at baseline
Tsai et al. (1)	China	12 weeks	Spesolimab 900 mg IV	Placebo	5	6	NA	3 (60%)	47.2 (10.0)	42.0 (11.1)	Randomized, double-blind, placebo-controlled (Effisayil 1 subgroup)	Chinese patients experiencing an active flare of Generalized Pustular Psoriasis (GPP)
Morita et al. (2)	Asia	12 weeks	Spesolimab 900 mg IV	Placebo	16	13	NA	10 (62.5%)	42.2 (11.6)	43.2 (9.2)	Randomized, double-blind, placebo-controlled (Effisayil 1)	Asian patients with flares of generalized pustular psoriasis (GPP)
Bachelez et al. (4)	Multinational (12 countries)	12 weeks	Spesolimab 900 mg IV	Placebo	35	18	NA	21 (60%)	43.2 (12.1)	42.6 (8.4)	Randomized, double-blind, placebo-controlled (Effisayil 1)	Adults ≥ 18 years with generalized pustular psoriasis flare (GPPG A* total ≥ 3 and pustulation subscore ≥ 2), diagnosed per JDA* criteria

*GPPGA, Generalized Pustular Psoriasis Physician Global Assessment. *ERASPEN, European Rare and Severe Psoriasis Expert Network. *JDA, Japanese Dermatological Association.

### Risk of bias assessment

**FIGURE 2:**
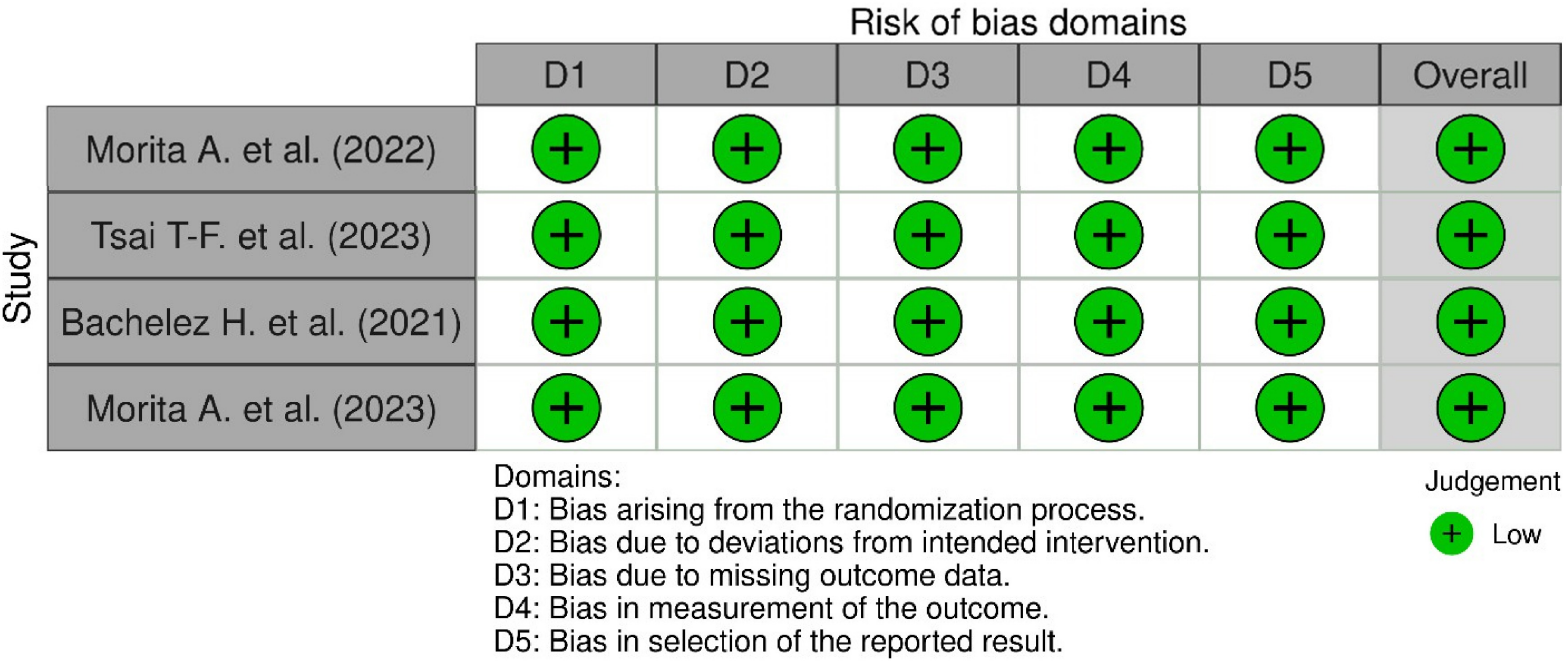
Risk of bias summary.

**FIGURE 3:**
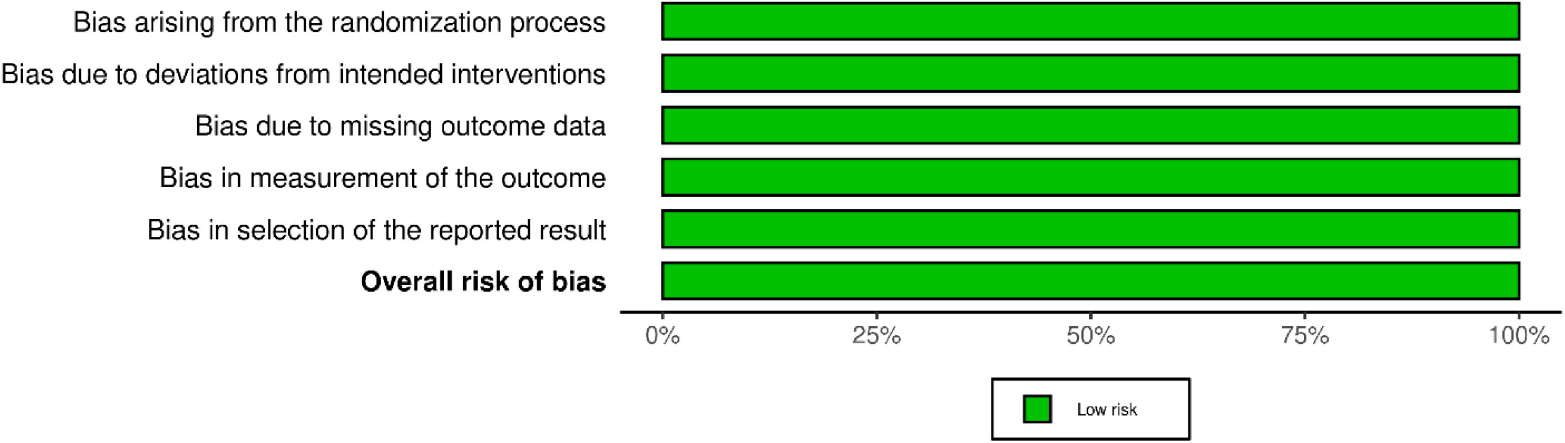
Risk of bias graph.

The included studies comprised the global Effisayil 1 trial (4), two subgroup analyses (1, 2), and the Effisayil 2 trial (3). All four studies were judged to have a low risk of bias across all five RoB 2 domains. Randomization was centrally generated with allocation concealment, prespecified stratification factors, and balanced baseline characteristics. All trials were double-blind and placebo-controlled, ensuring masking of participants, investigators, and study personnel. Outcome data were complete for ≥ 95–98% of participants, with missing data handled according to predefined methods. Primary outcomes were objective, standardized clinical assessments performed by blinded investigators using indistinguishable placebo preparations. Finally, all studies had publicly registered protocols and prespecified analyses, with no evidence of selective reporting. Overall, all trials demonstrated consistently low risk of bias across all domains. The results of the risk of bias assessment are presented in Figures 2, 3.

### Efficacy outcomes

#### GPPGA pustulation sub-score of 0

**FIGURE 4:**
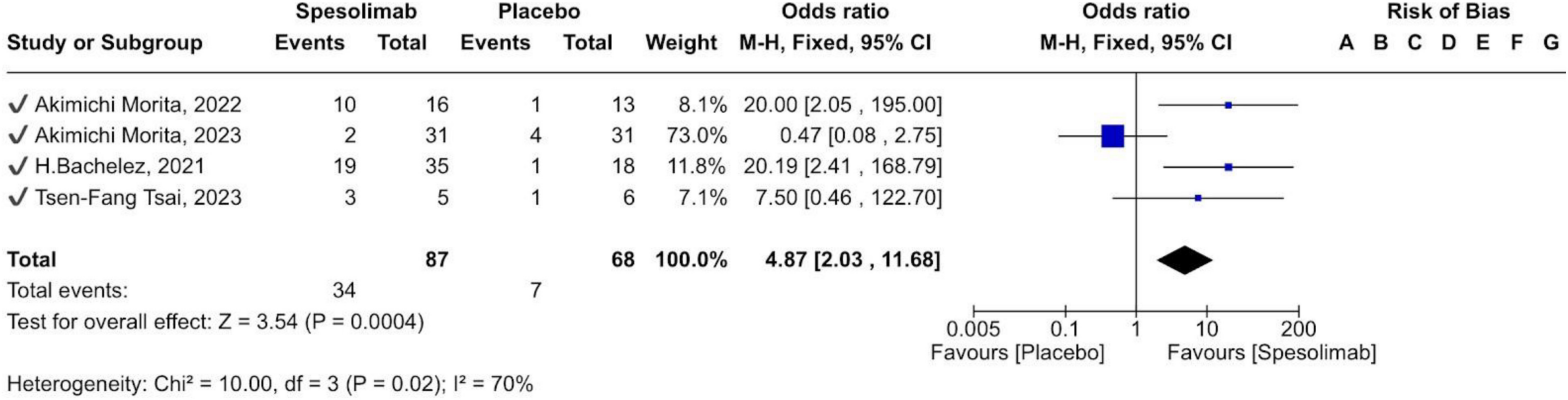
forest plot of (GPPGA) pustulation sub-score of 0.

**FIGURE 5:**
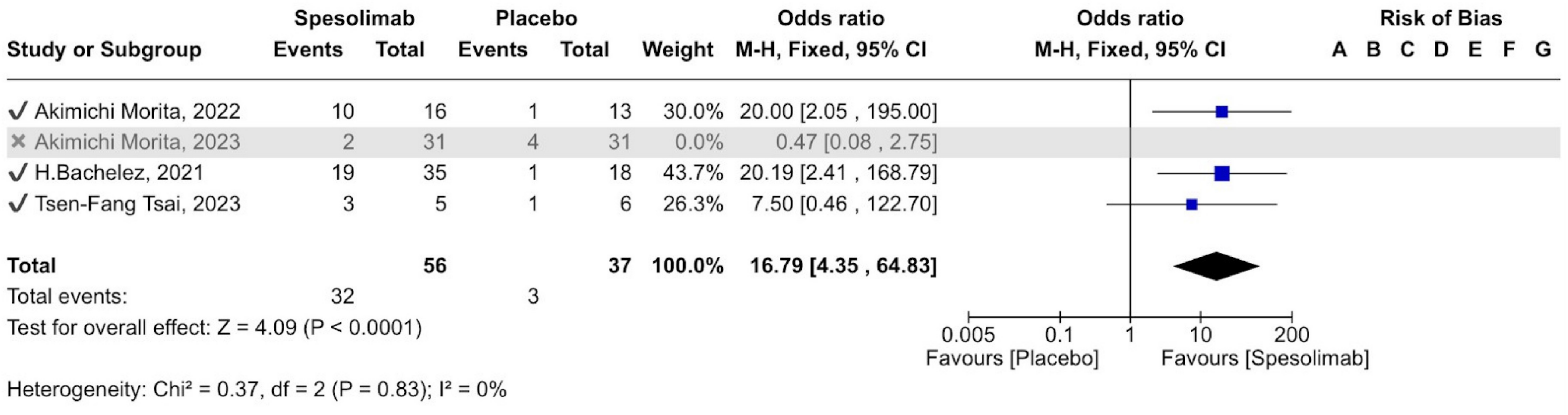
forest plot of (GPPGA) pustulation sub-score of 0 after sensitivity analysis.

Four RCTs evaluated the efficacy of spesolimab versus placebo in achieving a GPPGA pustulation sub-score of 0. Across all studies, 87 participants received spesolimab and 68 received placebo. The pooled results showed a statistically significant difference between groups [OR 4.87 (95% CI 2.03–11.68); *p* = 0.0004], indicating that spesolimab achieved nearly five-fold higher pustule clearance compared with placebo (Figure 4). Due to high heterogeneity (*I*^2^ = 70%), a sensitivity analysis was performed excluding the Akimichi Morita 2023 trial, which contributed most to variability. After exclusion, the odds ratio increased to 16.79 (95% CI 4.35–64.83; *p* < 0.0001), further confirming a superior efficacy of spesolimab in achieving a GPPGA pustulation sub-score of 0 in three of the four trials (Figure 5). Clinical heterogeneity may partly reflect differences in trial objectives and baseline status, including inclusion of Effisayil 2, a maintenance/prevention study enrolling patients who were clear/almost clear at baseline.

#### GPPGA total score of 0 or 1

**FIGURE 6:**
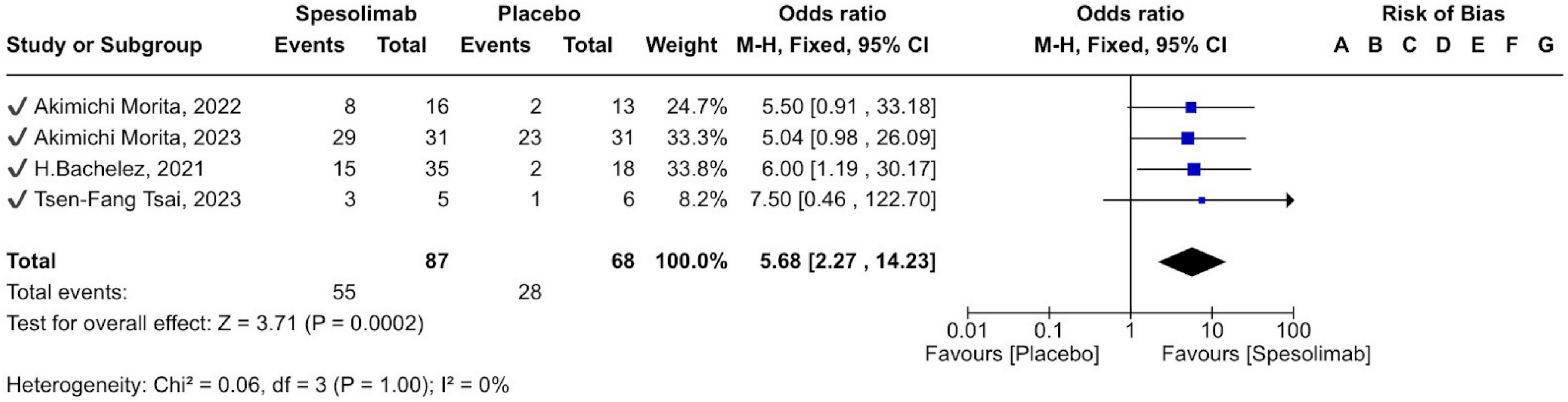
forest plot of (GPPGA) total score of 0 or 1.

The pooled meta-analysis demonstrated significant superiority of spesolimab over placebo in achieving a GPPGA total score of 0 or 1 [OR 5.68 (95% CI 2.27–14.23); *p* = 0.0002]. This indicates that patients treated with spesolimab were approximately 5.7 times more likely to reach near-complete or complete skin clearance than those receiving placebo (Figure 6).

#### Adverse events

**FIGURE 7:**
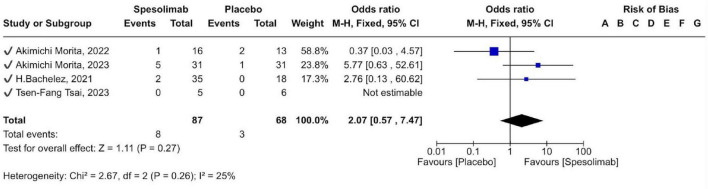
forest plot of adverse events (AE).

Adverse events were reported in 8 of 87 participants (9.2%) receiving spesolimab and 3 of 68 (4.4%) receiving placebo. Adverse events such as urinary tract infections, pyrexia, pain in extremities, and headache were evaluated in the analysis. Although adverse events were numerically more frequent in the spesolimab group, the difference was not statistically significant [OR 2.07 (95% CI 0.57–7.47); *p* = 0.27]. Overall, spesolimab demonstrated an acceptable safety profile with no significant increase in adverse event rates compared with placebo (Figure 7).

#### GRADE evidence assessment

The certainty of evidence for spesolimab was rated as moderate for efficacy outcomes and very low for any adverse events. Downgrades were applied for inconsistency, imprecision, and sparse data. For efficacy, spesolimab probably increases the proportion of patients achieving rapid pustule clearance and overall clinical improvement at Week 1 compared with placebo (moderate certainty evidence). Across the included four RCTs, 39.1% of participants receiving spesolimab achieved a GPPGA pustulation sub score of 0. Meanwhile participants receiving placebo only 10.3% achieved a GPPGA pustulation sub score of 0 (OR 4.87, 95% CI 2.03–11.68). In addition, 63.2% of participants taking spesolimab achieved a GPPGA total score of 0/1. In contrast, 41.2% of participants receiving placebo achieved a GPPGA total score of 0 or 1 (OR 5.68, 95% CI 2.27–14.23). For safety, in the four included trials, adverse events were uncommon, 9.2% with spesolimab in contrast to 4.4% with placebo (OR 2.07, 95% CI 0.57–7.47). However, the certainty of the safety estimate is very low. This is because of the very wide confidence interval and sparse data.

Overall, these GRADE judgments suggest with moderate confidence that spesolimab provides clinically meaningful short term improvement in GPP signs and symptoms. Meanwhile, spesolimab effect on short term adverse events remains highly uncertain. This highlights the need for larger and longer term trials. Detailed GRADE evidence profile is provided in Table 3.

**TABLE 3:** GRADE evidence profile.

Certainty assessment							No. of patients		Effect		Certainty	Importance
No. of studies	Study design	Risk of bias	Inconsistency	Indirectness	Imprecision	Other considerations	Spesolimab	Placebo	Relative (95% CI)	Absolute (95% CI)		
**GPPGA pustulation sub-score of 0.**												
4	Randomize d trials	Not serious	Serious	Not serious	Not serious	None	34/87 (39.1%)	7/68 (10.3%)	OR 4.87 (2.03–11.68)	260 more per 1,000 (from 90 more to 470 more)	⊕⊕⊕○ Moderate^a^	Critical
**GPPGA total score of 0 or 1**												
4	Randomize d trials	Not serious	Not serious	Not serious	Not serious	None	55/87 (63.2%)	28/68 (41.2%)	OR 5.68 (2.27–14.23)	238 more per 1,000 (from 133 fewer to 369 more)	⊕⊕⊕○ Moderate^b^	Critical
**Adverse events**												
4	Randomize d trials	Not serious	Not serious	Not serious	Very serious	None	8/87 (9.2%)	3/68 (4.4%)	OR 2.07 (0.57 to 7.47)	40 more per 1,000 (from 19 fewer to 203 more)	⊕○○○ Very low^c,d^	Important

CI, confidence interval; OR, odds ratio. ^a^Downgraded one level for inconsistency: substantial heterogeneity (*I*^2^ = 70%) and variation in effect sizes across the studies, although all point estimates favored spesolimab. ^b^Downgraded one level for study limitations related to small total sample size and imprecision typical of rare-disease evidence, despite consistent effect estimates and no statistical heterogeneity (*I*^2^ = 0%). ^c^Downgraded two levels for very serious imprecision: the confidence interval was wide and crossed both no effect and important harm thresholds (OR 0.57–7.47); optimal information size was not met. ^d^Downgraded one additional level for sparse data: adverse events were infrequent, with several studies reporting zero events, resulting in instability and uncertainty in the effect estimate.

## Discussion

GPP is a rare but potentially life-threatening inflammatory disease that poses substantial clinical challenges because of its unpredictable course and limited evidence-based treatment options (18). In this systematic review and meta-analysis, we pooled data from four randomized controlled trials and found that blocking the interleukin-36 (IL-36) receptor with spesolimab significantly improves pustule clearance and overall disease severity during acute flares compared with placebo. Patients receiving spesolimab were nearly five times more likely to achieve complete pustule clearance and showed numerically higher rates of near complete skin clearance without a significant increase in adverse events. These findings support the central role of IL-36 signaling in GPP pathogenesis and confirm that directly antagonizing this pathway can provide rapid and clinically meaningful relief for patients experiencing flares (11). Our results align with growing evidence from observational studies and early case series that suggest spesolimab leads to rapid improvements in GPP symptoms. For example, in a recent single-center retrospective study of seven patients, a median reduction of more than 63.6% in the Generalized Pustular Psoriasis Area and Severity Index was observed within the first three days of treatment, and all patients achieved a pustulation sub-score of zero by day 7 (19). Non-randomized reports generally describe early clinical improvement after spesolimab; however, these designs are susceptible to selection bias, regression to the mean, and confounding, and therefore primarily provide contextual information rather than comparative estimates. Consequently, these data suggest that spesolimab may offer the rapid disease control that patients and clinicians need during severe flares.

Comparative evidence indicates that therapies targeting other cytokine pathways have more variable or delayed effects in GPP. IL-23 inhibitors such as guselkumab have shown durable improvements in some patients but often require weeks to achieve meaningful disease control and may be better suited for maintenance therapy (20). Tumor necrosis factor–α inhibitors like infliximab or adalimumab have also been used off-label; while infliximab sometimes induces rapid pustule resolution, responses to adalimumab are inconsistent and relapse after discontinuation is common (21, 22). By contrast, spesolimab acts directly on the IL-36 receptor, a pivotal mediator of neutrophilic and keratinocytic activation in GPP, which may explain its rapid and reliable efficacy across trials (1–4). Because IL-36 signaling is implicated in neutrophilic inflammation in GPP, IL-36 receptor blockade represents a mechanistically targeted approach. Direct comparisons with other systemic options remain limited, and head-to-head trials or comparative effectiveness studies would be required to define its relative positioning (11).

In the included RCTs, adverse events were uncommon and did not differ statistically between groups, but the safety estimate was imprecise due to sparse events and short follow-up. Ongoing pharmacovigilance and longer trials are needed to better characterize rare and longer-term risks. These findings are consistent with previous reports showing that patients generally tolerate IL-36 receptor blockade well (23). Nevertheless, the rarity of GPP and the small sample sizes of existing trials mean that rare or long-term safety signals cannot be excluded. Real-world pharmacovigilance and registries will be essential to identify infrequent adverse events and to determine whether repeated dosing or chronic use leads to any new safety concerns.

Off-label/experimental use beyond GPP flares has also been described. A recent review summarized reports and early clinical data evaluating IL-36 receptor blockade with spesolimab in several inflammatory skin diseases in which IL-36 signaling may contribute to pathophysiology, including palmoplantar pustulosis, acrodermatitis continua of Hallopeau, hidradenitis suppurativa, pyoderma gangrenosum, and acute generalized exanthematous pustulosis (24). While these publications suggest potential activity in selected refractory cases, the evidence base is predominantly non-randomized and heterogeneous, with limited sample sizes and variable outcome definitions; therefore, these uses remain investigational and do not provide comparative estimates analogous to RCTs in GPP.

This review synthesizes the currently available randomized evidence on spesolimab for acute GPP flares; however, conclusions are limited by small sample sizes, potential non-independence across reports, heterogeneity in designs/dosing, and predominantly short follow-up. The pooled analysis enhances precision and strengthens the evidence supporting clinical decision for this rare and life-threatening condition. Despite the valuable findings in this study, the majority of patients were of Asian ethnicity, and follow-up periods were relatively short. Thus, such factors limit the generalizability of the results and preclude robust assessment of long-term outcomes, relapse rates, or the impact of repeated flares. Moreover, the high heterogeneity observed in the primary outcome underscores the need for further standardization of trial designs and outcome measures. A further limitation is that subgroup analyses from the Effisayil 1 program were included alongside the parent trial report. This may introduce non-independence and could artificially narrow confidence intervals; however, the direction of effect remained consistently favorable across the included reports. A key limitation is that Effisayil 2 was primarily a maintenance/prevention study with lower baseline disease activity (GPPGA 0–1) and a different primary endpoint (time to first flare) than acute-flare trials. This may limit the interpretability and applicability of pooled Week-1 clearance estimates to patients presenting with more severe acute flares. Future research should aim to address these gaps. Larger, multi-ethnic cohorts with longer follow-up are needed to confirm the durability of response and to evaluate safety over extended periods. Studies comparing spesolimab head-to-head with other targeted agents or exploring combination approaches could clarify the optimal positioning of IL-36 blockade within a comprehensive set of treatment options for GPP.

## Conclusion

This systematic review and meta-analysis provide the most comprehensive synthesis to date of randomized controlled trial data assessing spesolimab for acute flares of generalized pustular psoriasis. By directly inhibiting the IL-36 receptor, spesolimab leads to rapid clearance of pustules and significant improvements in disease severity without a significant increase in adverse events. Larger, longer-duration trials are needed to confirm durability of response, relapse prevention, and safety.

## Data Availability

The original contributions presented in the study are included in the article/Supplementary material, further inquiries can be directed to the corresponding author.
