# Supplementary material for: The efficacy and safety of spesolimab in patients with generalized pustular psoriasis flares: a systematic review and meta-analysis
Source: Front Med (Lausanne). 2026 Jan 15;12:1749320. doi: 10.3389/fmed.2025.1749320 (PMC12851960; doi:10.3389/fmed.2025.1749320)
Supplement: Supplementary file 1 [file Table_1.docx]

Supplementary Table S1. Non-randomized evidence on spesolimab for generalized pustular psoriasis (GPP) flares

| **Study (Year)** | **Country/Setting** | **Design / Study type** | **N (GPP flare episodes/patients)** | **Spesolimab regimen** | **Follow-up / assessment timepoints** | **Key reported outcomes (high level)** |
| --- | --- | --- | --- | --- | --- | --- |
| **Krefting et al. (2024)** [[1]](https://sciwheel.com/work/citation?ids=18479312&pre=&suf=&sa=0&dbf=0) | Germany | Compassionate Use Program (CUP); observational patient-level analysis | 12 patients | IV 900 mg at baseline; option for 2nd dose Day 8 | Baseline, Day 8, Week 4 | Clinical improvement reported by Day 8 and Week 4 using GPPGA and pustulation subscore; no drug-related AEs reported. |
| **Okada et al. (2025)** [[2]](https://sciwheel.com/work/citation?ids=18479313&pre=&suf=&sa=0&dbf=0) | Japan (single centre) | Retrospective real-world study (research letter) | 7 patients (reported in secondary summaries) | Real-world spesolimab use (IV dosing consistent with approved flare treatment) | Short-term post-treatment assessments | Rapid clinical improvement and tolerability reported; uncontrolled design. |
| **Rittig et al. (2025)** [[3]](https://sciwheel.com/work/citation?ids=18479315&pre=&suf=&sa=0&dbf=0) | Denmark | Case series; compassionate use prior to commercial availability | 3 patients | Spesolimab administered via compassionate use | Early post-treatment course | Rapid improvement across severe cases; descriptive/uncontrolled evidence. |
| **Ran et al. (2023)** [[4]](https://sciwheel.com/work/citation?ids=18479316&pre=&suf=&sa=0&dbf=0) | China | Case series / Letter to the Editor | 5 patients | Spesolimab used for acute flare | Week 0, Week 1, Week 4, Week 16 | Serial clinical scoring (e.g., GPPGA/GPPASI) with rapid response described; safety events documented; uncontrolled. |

This table is provided to summarize study types outside RCTs and is not used for pooled comparative inference.

**References**

[[1]    F. Krefting *et al.*, “Analysis of the German Compassionate Use Program on spesolimab in patients with generalized pustular psoriasis: evidence outside of clinical trials.,” *Eur. J. Dermatol.*, vol. 34, no. 6, pp. 643–650, Dec. 2024, doi: 10.1684/ejd.2024.4785.](https://sciwheel.com/work/bibliography/18479312)

[[2]    Y. Okada, M. Kamata, K. Hayashi, K. Sugiura, and Y. Tada, “Effectiveness and Safety of Spesolimab in Patients with Generalized Pustular Psoriasis: A Single-centre Retrospective Study.,” *Acta Derm. Venereol.*, vol. 105, p. adv42879, Feb. 2025, doi: 10.2340/actadv.v105.42879.](https://sciwheel.com/work/bibliography/18479313)

[[3]    A. H. Rittig, T. Bertelsen, S. D. W. Stave, and L. Iversen, “Early Real-Life Experience with Spesolimab in the Treatment of Generalized Pustular Psoriasis: A Case Series of Three Patients Treated in a Compassionate Use Program.,” *Case Rep. Dermatol.*, vol. 17, no. 1, pp. 376–381, Jul. 2025, doi: 10.1159/000547428.](https://sciwheel.com/work/bibliography/18479315)

[[4]    D. Ran *et al.*, “Rapid and sustained response to spesolimab in five Chinese patients with generalized pustular psoriasis.,” *Clin. Exp. Dermatol.*, vol. 48, no. 7, pp. 803–805, Jul. 2023, doi: 10.1093/ced/llad108.](https://sciwheel.com/work/bibliography/18479316)
